# Changes in Adrenergic and Cholinergic Receptors on Human Cerebral Vessels

**DOI:** 10.3390/ph19101518

**Published:** 2026-09-25

**Authors:** Maureen Gatherer, Christopher Brown, Laura Chinezu, Doina Manu, Anna Williams, Abigail Pendower, Juliette Ainslie, Lauren Walker, Johannes Attems, Louise Kelly, Scott Counts, Roxana Carare

**Affiliations:** 1 Faculty of Medicine, University of Southampton, Southampton SO16 6YD, UK; c.m.brown@soton.ac.uk (C.B.); aw2g20@southamptonalumni.ac.uk (A.W.); abbie.pendower@gmail.com (A.P.); julietteainslie1@gmail.com (J.A.); l.kelly@soton.ac.uk (L.K.); 2 Center for Advanced Medical and Pharmaceutical Research, “George Emil Palade” University of Medicine, Pharmacy, Science and Technology of Targu Mures, 540142 Targu Mures, Romania; laura_chinezu@yahoo.com (L.C.); doina.manu@umfst.ro (D.M.); 3 Translational and Clinical Research Institute, Newcastle University, Newcastle upon Tyne NE1 7RU, UK; lauren.walker@tees.ac.uk (L.W.); johannes.attems@ncl.ac.uk (J.A.); 4 Institute of Neuropathology and Neuromolecular Pathology, Medical University of Innsbruck, 6020 Innsbruck, Austria; 5 Department of Translational Neuroscience, College of Human Medicine, Michigan State University, Grand Rapids, MI 49503, USA; scott.counts@hc.msu.edu

**Keywords:** CAA, cerebrovascular, muscarinergic, cholinergic

## Abstract

**Background/Objectives**: Sporadic cerebral amyloid angiopathy (CAA) occurs due to a failure of clearance of amyloid-beta (Aβ) along the walls of capillaries and arteries. Soluble Aβ is eliminated from the brain along the basement membranes of capillaries and arteries as Intramural Periarterial Drainage (IPAD). The motive force for IPAD is provided by the spontaneous contractions of vascular smooth muscle cells, as vasomotion. Cerebrovascular smooth muscle cells receive adrenergic and cholinergic innervation from the Locus Coeruleus and the Nucleus Basalis of Meynert, which are compromised very early in Alzheimer’s disease (AD). Here, we tested the hypothesis that adrenergic and cholinergic receptors are histologically preserved in parenchymal and leptomeningeal blood vessels with CAA. **Methods**: Using immunohistochemistry, we examined the cerebrovascular distribution of muscarinic (M1, M2), nicotinic (α 7 nicotinic acetylcholine receptor), and adrenergic (α1b/2b) receptors in post-mortem brain tissue. We compared receptor distribution across CAA and non-CAA patients, as well as young and old patients without CAA. **Results**: We found significant differences in the receptor immunoreactivity of α7 nicotinic acetylcholine receptors, as well as α1b and α2b adrenergic receptors. However, there were no differences in the receptor immunoreactivity of M1 or M2 muscarinic receptors between patient groups. **Conclusions**: As IPAD depends on the functionality of the vascular smooth muscle cells to maintain the integrity of IPAD with aging, based on these histological findings, future therapeutic interventions could focus on agonists of α7 nicotinic acetylcholine and α1b/2b adrenergic receptors.

## 1. Introduction

Cerebral amyloid angiopathy (CAA) is a key feature of Alzheimer’s disease (AD) [1]. In CAA, there is deposition of amyloid-beta peptides (Aβ) in the walls of capillaries and arteries, and this occurs as a result of a failure of elimination of Aβ from the brain [2]. As the brain contains no conventional lymphatics, interstitial fluid and solutes, including Aβ, are normally eliminated along basement membranes within the walls of cerebral capillaries and arteries, known as intramural peri-arterial drainage (IPAD) pathways [3]. IPAD fails when arteries age and in individuals with the apolipoprotein (APO) ε4 genotype, resulting in the accumulation of insoluble Aβ as CAA [4,5]. IPAD is different from glymphatic flow of cerebrospinal fluid (CSF), which is represented by convective influx of CSF into the brain parenchyma along the periarterial compartment, followed by drainage along the walls of veins [6]. While there is uncertainty regarding the contribution of IPAD or glymphatic drainage to the clearance of Aβ, the distribution of insoluble Aβ in CAA suggests that failure of IPAD is key to the pathogenesis of CAA [7,8].

Recent modeling and experimental studies suggest that spontaneous contraction of cerebrovascular smooth muscle cells provides the motive force for IPAD, a process known as vasomotion [9,10]. Vasomotion occurs at a frequency of 0.1 Hz and is independent of cardiac pulsations [11]. Cerebral arteries receive adrenergic supply from the Locus Coeruleus (LC) and cholinergic innervation from the Nucleus Basalis of Meynert (NBM). Aging is associated with degeneration of both the LC and NBM, and the deposition of phosphorylated tau in these centers is a feature of AD [12,13]. In AD, the LC and NBM are affected early with well-characterized neuropathological features [14]. The adrenergic innervation of cerebrovascular smooth muscle cells from the LC and the cholinergic supply from the NBM represent attractive targets for enhancing IPAD. Vascular smooth muscle cells express adrenergic and cholinergic receptors on the cell membrane and within cells [15], but despite studies showing widespread expression of adrenergic and cholinergic receptors in the brain [16,17,18], their exact distribution in the walls of cerebral arteries is uncertain.

Several Aβ species have been described, and one such species, termed pyroglutamylated Aβ (pAβ), has been suggested to play a crucial role in age-associated neurodegeneration. Recent evidence indicates that pAβ, which is generated by N-terminal truncation of full-length Aβ and subsequent cyclization of N-terminal glutamate by glutaminyl cyclase [19,20], is increased in AD, constituting 25% of the total plaque load. A similar amount of non-modified Aβ is apparently clinically undetectable in aged controls [21,22]. pAβ has recently been observed in CAA [23], but a full histopathological study has not yet been conducted to characterize the pattern of pAβ depositions in cerebral blood vessels and how this is related to adrenergic and cholinergic receptors. As CAA is more frequent in the occipital cortex, we utilized sections from this region throughout the present study [24].

Here, we compare the distribution of select adrenergic and cholinergic receptors on the walls of occipital cerebral arteries; the α7 nicotinic acetylcholine receptor (α7nAChR), M1 muscarinic acetylcholine receptor (M1mAChR), M2 muscarinic acetylcholine receptor (M2mAChR), M3 muscarinic acetylcholine receptor (M3mAChR), α 1B adrenergic receptor (ADRA1B), α 2A adrenergic receptor (ADRA2A), and α 2B adrenergic receptor (ADRA2B); across CAA and non-CAA patients, as well as young and old patients without CAA. We also characterized the association between pyroglutamylated Aβ and vessels expressing these receptors.

## 2. Results

We examined the vascular distribution of adrenergic and cholinergic receptors in samples of human occipital cortex from three groups of post-mortem donors: healthy young patients were <60 years old with no significant neuropathology, healthy old patients were ≥60 years old with no AD or CAA pathology, and CAA patients were ≥60 years old with CAA diagnosed by neuropathologists at Newcastle University. Diagnostic group age distributions can be seen in Figure S1.

All adrenergic and cholinergic receptors were detected throughout the brain parenchyma and blood vessels using immunohistochemistry, except for the cholinergic M3 receptor, which was not detected in blood vessels of any patient group. As the purpose of this study was to characterize the distribution of the receptors on blood vessels, M3 was not pursued further.

Primary antibodies used for immunohistochemistry were validated using Western blot. Antibodies for α7nAChR, M1mAChR, M2mAChR, ADRA1B, and ADRA2B bound to proteins in primary human smooth muscle cell lysates, but ADRA2A antibodies did not (Figure S2). As a result, ADRA2A was excluded from the analysis.

Immunostained brain sections were surveyed at low magnification, and a microarray of 10 × 0.5 mm^2^ adjacent ROIs in the cortical grey matter and leptomeningeal tissue at the brain surface or sulci was captured from each patient (Figure S3). Within each set of ROIs, the first five vessels to meet the criteria for analysis (diameter ≥ 15 µm, transverse section) were chosen for high-magnification imaging and quantification. We measured the immunoreactivity for each receptor as a percentage of the total area of blood vessels in transverse section and compared the distribution of each receptor across patient age and CAA pathology status using linear mixed effects models. Raw and Benjamini–Hochberg false discovery rate-adjusted (BH-FDR) omnibus probabilities for all five receptor models are presented in Table 1. Statistical significance in the text is based on BH-FDR-adjusted q-values.

After BH-FDR correction, significant Group × Anatomical Region interactions were retained for α7nAChR, ADRA1B, and CHRM1 (all q < 0.05). A significant main effect of diagnostic group was retained for α7nAChR and ADRA2B.

### 2.1. α7 Nicotinic Acetylcholine Receptors

α7nAChR exhibited the greatest variability of the receptors we investigated. Statistically significant differences in α7nAChR immunoreactivity were observed in both vascular compartments (Figure 1). Age-adjusted linear mixed-effects modeling demonstrated a significant overall effect of diagnostic group (F_2,25.92_ = 7.04, q = 0.0099) and anatomical region (F_1,269.94_ = 28.56, q < 0.001) on α7nAChR immunoreactivity (Figure 1h). Age was independently associated with α7nAChR immunoreactivity (F_1,25.9_ = 9.14, *p* = 0.006). However, the effects of diagnostic group and the group × anatomical region interaction remained significant after age adjustment (F_2,269.94_ = 11.72, q < 0.001). Within leptomeningeal vessels, the age-adjusted estimated marginal mean was significantly lower in CAA than in healthy older donors (adjusted mean difference 27.72, 95% CI 14.53 to 40.92; Bonferroni-adjusted *p* < 0.001). No other within-region group comparisons were statistically significant after Bonferroni correction.

In the leptomeninges, group differences were also evident. Both healthy young and healthy old groups exhibited significantly greater immunostaining compared to CAA (young vs. CAA: mean difference = 31.52%, 95% CI 15.48 to 47.55, *p* < 0.0001; old vs. CAA: mean difference = 28.31%, 95% CI 12.27 to 44.35, *p* = 0.0002). However, there was no significant difference between healthy young and healthy old (mean difference 3.205%, 95% CI −12.83 to 19.24, *p* = 0.8803).

Comparisons between tissue compartments revealed a selective compartmental effect in the healthy old group, where parenchymal immunoreactivity was significantly lower than leptomeningeal levels (mean difference = −20.86%, 95% CI −34.21 to −7.52, *p* = 0.0028). No significant parenchyma–leptomeninges differences were observed in healthy young or CAA groups (*p* = 0.428 and *p* = 0.836, respectively).

These findings indicate that α7nAChR immunoreactivity is reduced in CAA compared to healthy controls, with the reduction most pronounced relative to young controls and evident in both vascular compartments. Additionally, age-related differences are compartment-specific, with reduced parenchymal immunoreactivity in older individuals relative to leptomeninges.

### 2.2. α 1B Adrenergic Receptors

Age-adjusted linear mixed-effects modeling identified a significant interaction between diagnostic group and anatomical region for ADRA1B immunoreactivity (F_2,265.10_ = 12.57, q < 0.001; Figure 2). The main effects of diagnostic group (F_2,26.09_ = 2.33, q = 0.196) and anatomical region (F_1,265.11_ = 1.88, q = 0.215) were not statistically significant.

Bonferroni-adjusted simple comparisons showed that, within parenchymal vessels, estimated ADRA1B immunoreactivity was higher in healthy young donors than in healthy older donors (adjusted mean difference 40.63, 95% CI 2.86 to 78.40, *p* = 0.032) and CAA donors (adjusted mean difference 41.71, 95% CI 6.50 to 76.92, *p* = 0.016). Healthy older and CAA donors did not differ within parenchymal vessels (adjusted mean difference 1.08, 95% CI −18.07 to 20.22, *p* = 1.000). No between-group comparisons were statistically significant within leptomeningeal vessels (all Bonferroni-adjusted *p* ≥ 0.728). These findings indicate that group-associated differences in ADRA1B immunoreactivity vary according to anatomical region.

### 2.3. α2B Adrenergic Receptors

Age-adjusted linear mixed-effects modeling demonstrated a significant overall effect of diagnostic group on ADRA2B immunoreactivity (F_2,26.01_ = 6.90, q = 0.0099; Figure 3). Neither the main effect of anatomical region (F_1,266.04_ = 0.91, q = 0.342) nor the group × anatomical region interaction (F_2,266.04_ = 2.35, q = 0.097) was statistically significant. Bonferroni-adjusted region-stratified comparisons showed lower ADRA2B immunoreactivity in CAA than in healthy young donors in parenchymal vessels (adjusted mean difference 42.38, 95% CI 3.68 to 81.08, *p* = 0.028) and leptomeningeal vessels (adjusted mean difference 49.45, 95% CI 10.73 to 88.16, *p* = 0.009). CAA also showed lower immunoreactivity than healthy older donors in leptomeningeal vessels (adjusted mean difference 28.82, 95% CI 7.61 to 50.04, *p* = 0.005). These findings indicate an overall association between diagnostic group and ADRA2B immunoreactivity, but do not provide sufficient evidence that the group effect differs between anatomical regions.

### 2.4. Other Receptors

LMM analysis revealed a significant BH-FDR-adjusted main effect of anatomical region (q < 0.001) and group × region interaction (q < 0.001) for M1mAChR, but the main effect of diagnostic group was not significant (Figure 4a). None of the main effects or interactions for M2mAChR were significant (Figure 4b).

M2mAChR and ADRA1B did not demonstrate significant effects across patient group or anatomical compartment, suggesting that levels of these receptors are not altered by age or CAA pathology in either parenchymal or leptomeningeal vessels (Figure 4b).

### 2.5. Immunofluorescence

For receptors that showed significant differences between patient groups, we used immunofluorescence to co-label target receptors with anti-αSMA to confirm vessel identity and anti-pAβ to qualitatively examine the special relationship between these receptors and pAβ in CAA cases.

α7nAChR fluorescent staining was fine and punctate within the arteriolar perivascular unit, in both parenchymal and leptomeningeal arterioles, and appeared to be reduced in the CAA patient group. α7nAChR staining was qualitatively closely associated with smooth muscle cells, which can be identified by continuous and circumferential αSMA staining of 10–30 µm thickness around cerebral blood vessels (Figure 5). pAβ was not observed in α7nAChR-positive arterioles in the parenchyma but was clearly present in α7nAChR-positive leptomeningeal arterioles of CAA individuals (Figure 5).

The ubiquitous expression of adrenergic receptors in the brain was evident by the presence of extensive ADRA2B-positive cells in the parenchyma; however, ADRA2B staining in the vessel wall was finer and more punctate in appearance. CAA patients appeared to have less ADRA2B staining than healthy old and healthy young individuals, which correlated with the presence of pAβ immunoreactivity in both the parenchymal and leptomeningeal compartments in CAA patients (Figure 6).

## 3. Discussion

This study demonstrates the histological preservation of adrenergic receptors ADRA1B and ADRA2B on the walls of parenchymal and leptomeningeal blood vessels from aged and CAA in the occipital lobe of postmortem human brains. In a previous study, we demonstrated the histological preservation of ADRA1A with aging and in CAA [25]. The percentage area immunostained for α7nAChR was significantly reduced in aging and CAA, but nevertheless, the receptor was still present in parenchymal and leptomeningeal vessels. We observed pyroglutamated Aβ deposited in the walls of blood vessels in young cases, suggesting that this form of Aβ may be an earlier form that is deposited when clearance mechanisms fail.

The overall pattern of reduction in the immunohistochemical representation of the adrenergic and cholinergic receptors with aging and in CAA probably further enhances the loss of innervation of the cerebrovascular mural cells. This will most likely have an impact on both perfusion of the brain as well as clearance of fluids and solutes.

Experimental studies using immunotoxins that resulted in cortical cholinergic deafferentation in wild-type rabbits led to endogenous deposition of Aβ in the walls of cerebral arteries [26]. More recently, experimental destruction of the Locus Coeruleus in a transgenic Alzheimer’s model of rats demonstrated a worsening of amyloid angiopathy [27]. Taken together, these studies demonstrate that both cholinergic and adrenergic supply are necessary for the prevention of CAA.

Radiological studies demonstrate that in AD there is a reduction in the size of both the LC and NBM, suggesting that both cholinergic and adrenergic input to the arterial smooth muscle cells are impaired [28,29]. Recent studies show that depletion of NBM results in long-term vasodilation of small arterioles and reduced diameter of capillaries, suggesting thus an alteration of vasomotor dynamics which would likely impact IPAD [29]. The results from the present study show the distribution of pyroglutamylated Aβ in the walls of arteries adjacent to smooth muscle cells and their adrenergic and cholinergic receptors. However, although demographic data were available, medication history of the donors was not. This would have influenced receptor presence and represents a limitation. In agreement with previous studies, in areas where there is deposition of pAβ, the distribution of smooth muscle actin is low, and there are fewer receptors [30], which could reflect the loss of smooth muscle cells as the deposition of pyroglutamylated Aβ in the walls of arteries increases.

Therapeutic attempts using acetylcholinesterase inhibitors only have limited effect in early stages of AD, suggesting that there needs to be a baseline level of acetylcholine in the extracellular spaces of the brain for their efficiency. Our study supports future studies testing the functionality of α7 nicotinic acetylcholine receptors, selected α1 and α2 adrenergic receptors, as well as the effects of agonists of α adrenergic receptors in combination with agonists of α7 nicotinic acetylcholine receptors towards facilitating IPAD. There are already adrenergic agonists in clinical use for treatment of attention-deficit/hyperactivity disorder: clonidine and dexmedetomidine act upon adrenergic receptors α2, with dexmedetomidine also increasing glymphatic influx [31,32]. Agonists and various allosteric modulators of α7 nicotinic acetylcholine receptors have been tested with variable results: A-582941, GTS-21, ABT-107, PNU-282987, EVP-6124, PNU-120596 [33]. However, clinical trial results using α7 nicotinic acetylcholine agonists and positive allosteric modulators have failed to match pre-clinical promise due to differences in receptor desensitization, poor pharmacokinetics (including variable results in smokers), and limitations in translational models [34]. Future efforts for facilitating IPAD in CAA and Alzheimer’s disease could encompass a combination therapy of agonists or partial allosteric modulators of α-adrenergic as well as α7nAChRs. Finally, through the findings of this study, we emphasize the importance of human neuropathology research and the valuable postmortem human brain tissue resources.

## 4. Materials and Methods

### 4.1. Human Tissue

Human brain tissue samples for this study were obtained from Edinburgh Sudden Death Tissue Brain Bank (Ethics REC 16/ES/0084) and Newcastle Brain Tissue Resource (Ethics REC 08/H0906/136 + 5).

We obtained formalin fixed, paraffin embedded (FFPE) sections of human occipital cortex from three groups of post-mortem donors: healthy young patients were <60 years old (*n* = 28, mean age 37.9) with no significant neuropathology, healthy old patients were ≥60 years old *(n* = 24, mean age 81.7) with no AD or CAA pathology, and CAA patients were ≥60 years old (*n* = 28, mean age 80.4) with CAA diagnosed by neuropathologists at Newcastle University. The mean ages of patients in healthy old and CAA groups were not significantly different (Figure S1). Detailed demographic data for individual patients can be found in Table S1.

Edinburgh Sudden Death Tissue Brain Bank and Newcastle Brain Tissue Resource obtained consent from all participants for the use of brain tissue in studies of neurodegenerative disease. All procedures involving post-mortem human tissue were in accordance with the ethical standards of the University of Southampton Research Ethics Policies, the 1964 Helsinki Declaration and its later amendments, and the United Kingdom Human Tissue Act 2004. This study was performed under the ethics approvals from East of Scotland Research Ethics Service (EoSRES) REC 21/ES/0087 and North East—Newcastle and North Tyneside Research Ethics Committee, REC 24/NE/0012.

### 4.2. Immunohistochemistry

FFPE sections (10 µm thick) were dewaxed by heating at 60 °C for 15 min and immersion in xylene, then rehydrated through graded alcohols for 3 min each. Endogenous peroxidase activity was quenched by incubation in 3% hydrogen peroxide in phosphate-buffered saline (PBS) for 15 min. Antigens were unmasked using heat-induced epitope retrieval (HIER) in citrate buffer (10 mM, pH 6) for 25 min in a microwave. For some primary antibodies, an additional pepsin digestion epitope retrieval step was performed (1% pepsin in 0.2 M HCl for 3 min at 37 °C).

Following epitope retrieval, sections were blocked in 15% normal goat serum (Merck Life Science, Gillingham, UK, #G9023) in PBS plus 0.1% Triton X-100 (PBS-T) for 1 h, then incubated overnight at 4 °C with primary antibodies (Table 2).

The following day, the sections were incubated for 1 h with a species-appropriate biotinylated goat anti-rabbit secondary antibody in PBS-T, then with Vectastain avidin–biotin complex (2BScientific, Kirtlington, UK, #PK-4000) at 1:200 dilution in PBS-T for 1 h. Antibody complexes were detected using substrate 3,3′-diaminobenzidine (DAB) with nickel enhancement (, 2BScientific, Kirtlington, UK #SK-4100). Finally, sections were dehydrated through graded alcohols, cleared with xylene, and mounted with glass coverslips using DPX mounting medium. Secondary-only and isotype controls were performed for each primary antibody used.

Imaging was performed with an Olympus BX51 dot slide microscope fitted with an Olympus CC12 microscope camera using the tissue microarray function. Overview images captured at 2× magnification were used to select 0.5 mm^2^ adjacent ROIs in the cortical grey matter and leptomeningeal tissue at the brain surface or sulci (Supplementary Figure S1). ROIs were then scanned at 20× magnification, and the first 5 non-capillary vessels (≥15 µm in diameter) in transverse section were selected from each of the brain parenchyma and leptomeninges for analysis.

### 4.3. Immunofluorescence

Qualitative triple immunofluorescence was used to determine the relationship of each receptor with respect to the presence of pyroglutamylated Aβ (pAβ) and smooth muscle actin (SMA). Tissue was processed as for immunohistochemistry up to the addition of primary antibody. PBS was substituted with Tris-buffered saline (TBS) to improve antibody permeability. pAβ detection required a further antigen retrieval step of 98% formic acid incubation for 3 min at room temperature immediately following rehydration.

Triple immunofluorescence required each cholinergic/adrenergic receptor primary antibody to be incubated overnight in conjunction with anti-SMA as well as anti-pAβ (Table 1). Following overnight incubation at 4 °C, sections were incubated in secondary antibodies, goat anti-mouse IgG1 Alexa FluorTM 647 together with goat anti-rabbit IgG, Alexa Fluor™ 555, for 1 h at room temperature, protected from light. Tissue autofluorescence was quenched via incubation with Sudan black (Merck Life Science, Gillingham, UK, #199664 1% *w*/*v* in 70% industrial methylated spirit). Slides were mounted using Mowiol aqueous mounting medium (, Merck Life Science, Gillingham UK, #81381) and stored at 4 °C until imaging.

Fluorescently labeled slides were imaged using a Leica TCS SP8 laser scanning confocal microscope fitted with Leica Application Suite X v3.5.23225.7. Images were captured using sequential scanning mode to avoid crosstalk, and false colors were applied to the lasers (Table 3). Optical sections were obtained every 4 µm, and maximum projection was applied to the Z-stack. The optical zoom function was utilized to show finer detail. Finally, images were exported as TIFF files.

### 4.4. Western Blotting

Human brain vascular smooth muscle cells (HBVSMCs) were purchased from Sciencell (Carlsbad, CA, USA, #1100) and grown according to the manufacturer’s instructions. Cells were maintained in specialized smooth muscle cell media obtained from Sciencell (#1101). Cultures were seeded at 1 × 10^6^ in a T75 flask coated with poly-L-lysine (15 µg/mL) (Sciencell, Carlsbad, CA, USA #0413,) and maintained in a humidified environment (5% CO_2_/95% air) at 37 °C until 90% confluent. Cells were washed in 1% PBS, then detached following incubation with 0.25% trypsin for 3 min. Cells were pelleted by centrifugation at 300× *g* and washed in ice-cold PBS before being re-pelleted and resuspended in 1ml of ice-cold RIPA buffer (Thermo Fisher Scientific, Waltham, MA, USA, #89900) containing 1 × Halt Protease and Phosphatase Inhibitor Cocktail (Thermo Fisher Scientific; Cat No. 78440).

Cell lysates were combined with Laemmli sample buffer (Bio-Rad, Hercules, CA, USA, #1610737) and 5% (*v*/*v*) 2-mercaptoethanol and loaded onto 12% mini-protean TGX gels (Bio-Rad, Hercules, CA, USA, #456-1044). Electrophoresis was conducted in Tris-Glycine-SDS buffer at 300 V for 20 min. Separated proteins were transferred to nitrocellulose membranes in Tris-Glycine buffer with 20% (*v*/*v*) methanol at 100 V for 30 min on ice. Membranes were blocked with 1% (*w*/*v*) milk/TBST solution, then probed with primary antibodies for all six receptors at 1:200 dilution. Bands were visualized with goat anti-rabbit IRDye 680 (LICORbio, Lincoln, NE, USA, #926-68071) and an Odyssey gel imaging system (LICORbio, Lincoln, NE, USA).

### 4.5. Image Processing

For both parenchymal grey matter and leptomeningeal vessels, 5 vessels per patient (10 patients per group) were analyzed using ImageJ 1.54g software. The first 5 non-capillary vessels (≥15 µm in diameter) in transverse section were selected for analysis. The quantification was not blinded.

A macro was used to create a mask of the vessel wall by removing the background and lumen (Figure S4). The analyze particles tool was used to measure the area of the vessel mask in pixels, then the number of pixels above a pre-defined threshold (Figure S5). The number of pixels above the threshold was divided by the area of the vessel mask and multiplied by 100 to give a figure for the percentage of the vessel mask containing immunoreactivity for each receptor (% area).

Thresholds were determined manually and applied across all micrographs from each receptor group. Due to differences in staining intensity and antibody penetration between leptomeningeal and parenchymal vessels, region-specific intensity thresholds were applied during image segmentation to ensure accurate detection within each vascular compartment (Table S2). Five technical replicates were averaged to give a single % area value for each patient. These values were exported to IBM SPSS Statistics 32.0.0 software for statistical analysis and GraphPad Prism 11.0.2 for data visualization.

### 4.6. Statistical Analysis

Five receptor outcomes were analyzed using separate linear mixed-effects models. Diagnostic group (young vs. old vs. CAA), anatomical region (parenchyma vs. leptomeninges), and the diagnostic group x anatomical region interaction were included as fixed effects, with patient identifier as a random intercept. Age was included as a covariate. The normality of residuals was validated using skewness and kurtosis measurements, histograms, and QQ plots.

To account for multiplicity across receptor outcomes, Benjamini–Hochberg false-discovery-rate correction (BH-FDR) was applied separately to the five omnibus *p*-values for diagnostic group, anatomical region, and the diagnostic group × anatomical region interaction. FDR-adjusted q-values below 0.05 were considered statistically significant. Within each receptor model, pairwise comparisons of estimated marginal means were conducted using the Bonferroni method on age-adjusted data. The alpha level for pairwise comparisons was also set at 0.05.

The results of linear mixed-effects models are presented as mean and 95% confidence intervals.

## 5. Conclusions

The spontaneous contraction of cerebral artery SMCs drives the clearance of soluble amyloid-β via the IPAD pathway (9, 10). Nuclei projecting to the adrenergic and cholinergic receptors on these cells are affected early in AD (14); therefore, we aimed to characterize the distribution of select receptors and examine their association with pyroglutamated amyloid-β in histological post-mortem samples from patients with CAA, a hallmark of AD, healthy old individuals, and healthy young individuals.

We found that the adrenergic receptors ADRA1B, ADRA2B, and cholinergic receptors α7nAChR, M1mAChR, and M2mAChR were all histologically observed in both leptomeningeal and parenchymal vessels. However, there were significant differences between some of the receptors and patient group/anatomical compartment. The most notable differences were demonstrated by cholinergic α7nAChR and adrenergic ADRA1B and ADRA2B receptors.

Pyroglutamated Aβ was predominantly seen in the CAA patient group and was spatially associated with the SMCs.

These findings may have implications for future therapeutic investigation, paving the way to the development of novel therapeutic regimens targeting the adrenergic and cholinergic cerebrovascular receptors. Future work could explore the functionality of the receptors, potential for augmentation, and thus help to maintain the integrity of the IPAD pathway.

## 6. Patents

This work was included in the patent application number GB2610028.9 from the University of Southampton, entitled “Combination therapy for Alzheimer’s disease and other cerebral amyloid angiopathy associated diseases” filed on 29 April 2026.

## Figures and Tables

**Figure 1:**
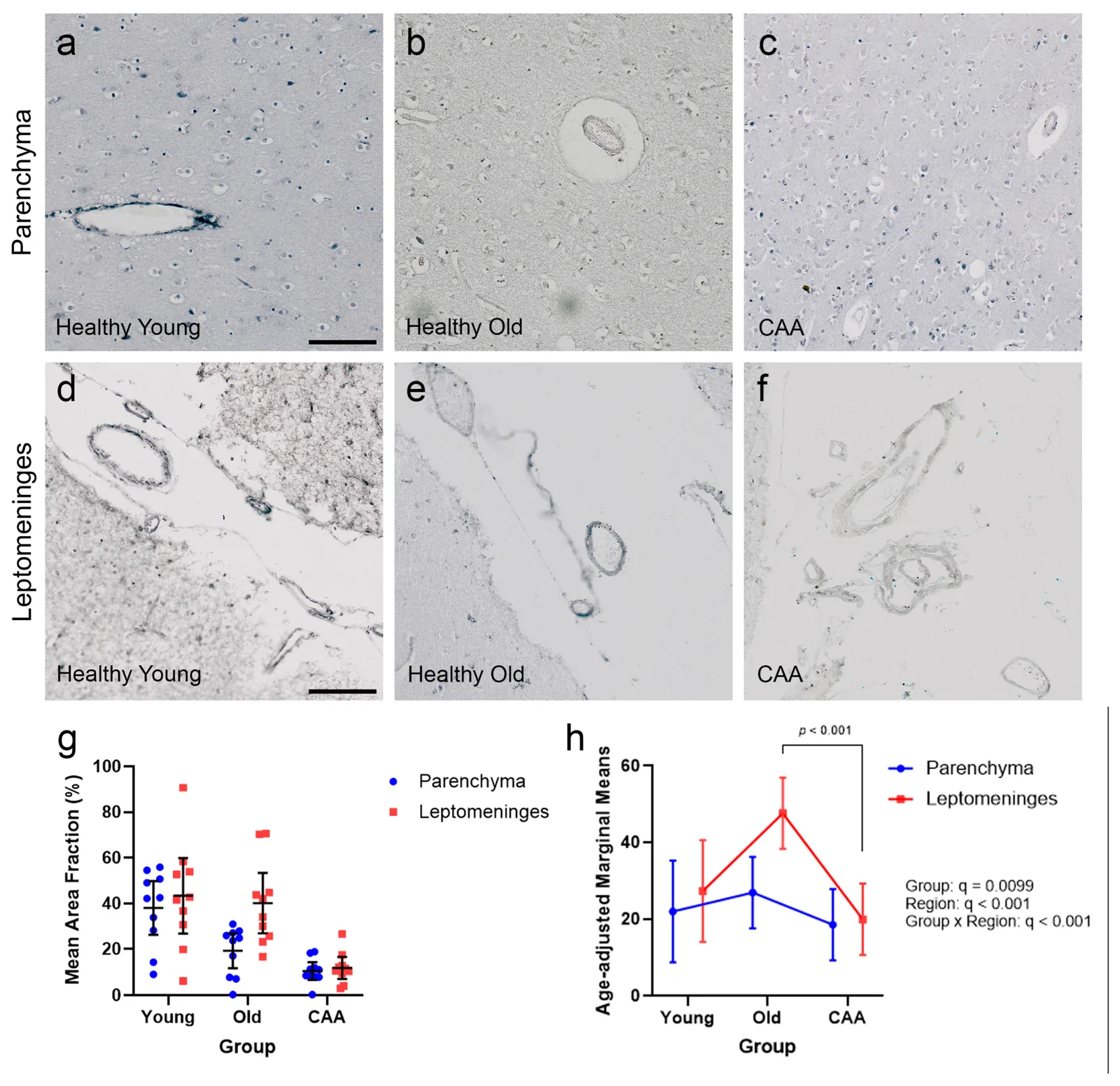
α7nAChR immunoreactivity is reduced in leptomeningeal vessels of CAA patients. Representative micrographs demonstrating immunoreactivity of α7nAChR in the walls of blood vessels in the brain parenchyma of healthy young (**a**), healthy old (**b**), and CAA patients (**c**), and leptomeningeal vessels of healthy young (**d**), healthy old (**e**), and CAA patients (**f**). Scale bars = 100 µm. (**g**) Patient-level area fraction values. Each point represents the mean area fraction across five vessels selected from the indicated anatomical region of one donor; *n* = 10 donors per diagnostic group. (**h**) Interaction plot showing age-adjusted estimated marginal means and 95% confidence intervals from a linear mixed-effects model containing diagnostic group, anatomical region, age, and the group × anatomical region interaction as fixed effects, with donor identifier as a random intercept. The main effect of diagnostic group was significant following BH-FDR correction (q = 0.0099), as was the anatomical region effect (q < 0.001), and the group × anatomical region interaction (q < 0.001). The confounding effect of age was also significant (*p* = 0.006). Bonferroni-adjusted within-region comparisons identified differences between healthy old and CAA donors in leptomeningeal vessels (*p* < 0.001). No other pairwise comparisons were significant.

**Figure 2:**
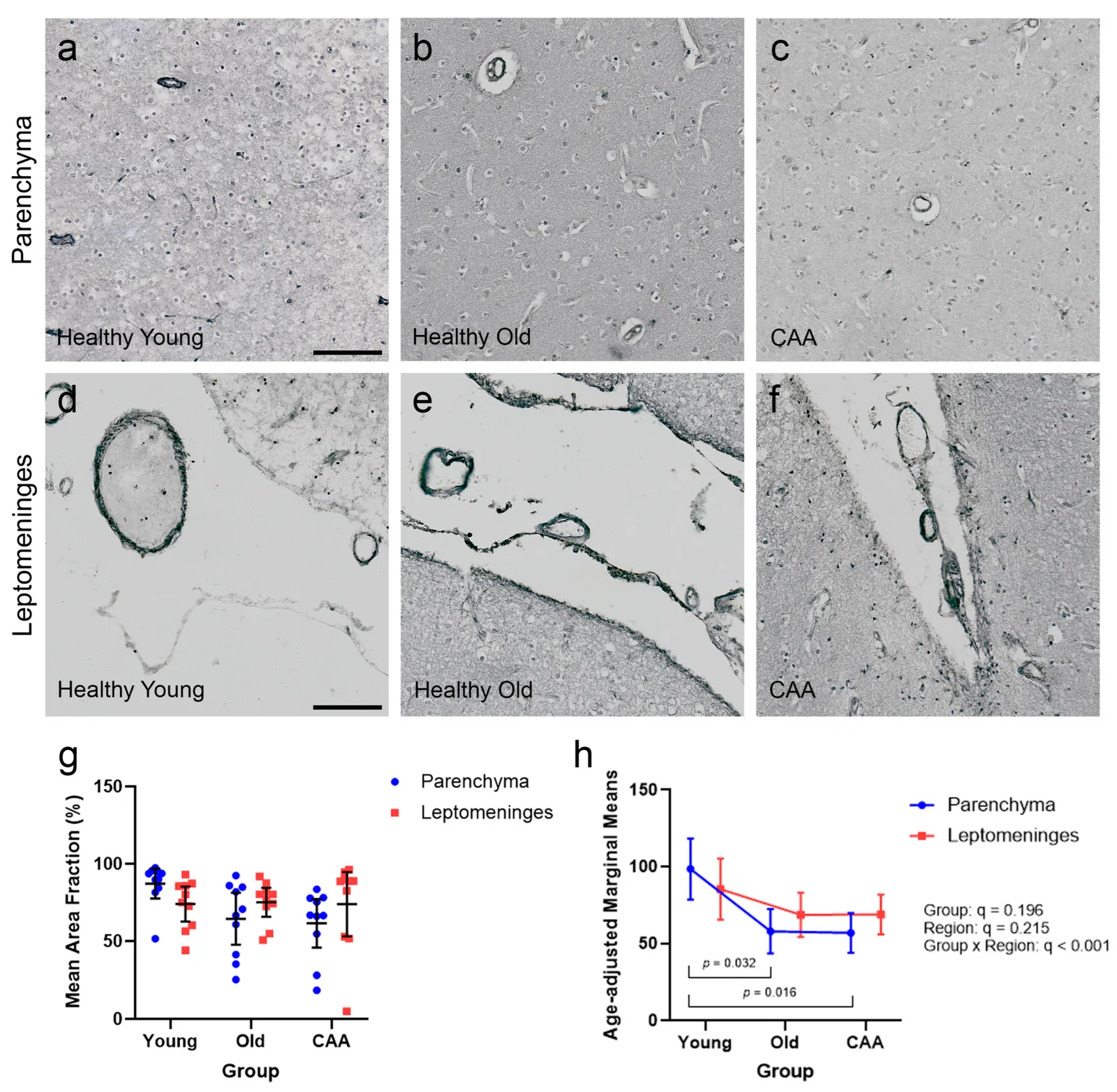
ADRA1B receptor immunoreactivity in parenchymal vessels is reduced in aging. Representative micrographs demonstrating immunoreactivity of ADRA1B in the walls of blood vessels in the brain parenchyma of healthy young (**a**), healthy old (**b**), and CAA patients (**c**), and leptomeningeal vessels of healthy young (**d**), healthy old (**e**), and CAA patients (**f**). Scale bars = 100 µm. (**g**) Patient-level area fraction values. Each point represents the mean area fraction across five vessels selected from the indicated anatomical region of one donor; *n* = 10 donors per diagnostic group. (**h**) Interaction plot showing age-adjusted estimated marginal means and 95% confidence intervals from a linear mixed-effects model containing diagnostic group, anatomical region, age, and the group × anatomical region interaction as fixed effects, with donor identifier as a random intercept. The diagnostic group × anatomical region interaction was significant (q < 0.001); the main effects of diagnostic group (q = 0.199) and anatomical region (q = 0.215) were not significant. Within parenchymal vessels, ADRA1B immunoreactivity was higher in healthy young donors than in healthy older donors (*p* = 0.032) and CAA donors (*p* = 0.016). No comparisons were significant within leptomeningeal vessels. Pairwise comparisons were adjusted using the Bonferroni method.

**Figure 3:**
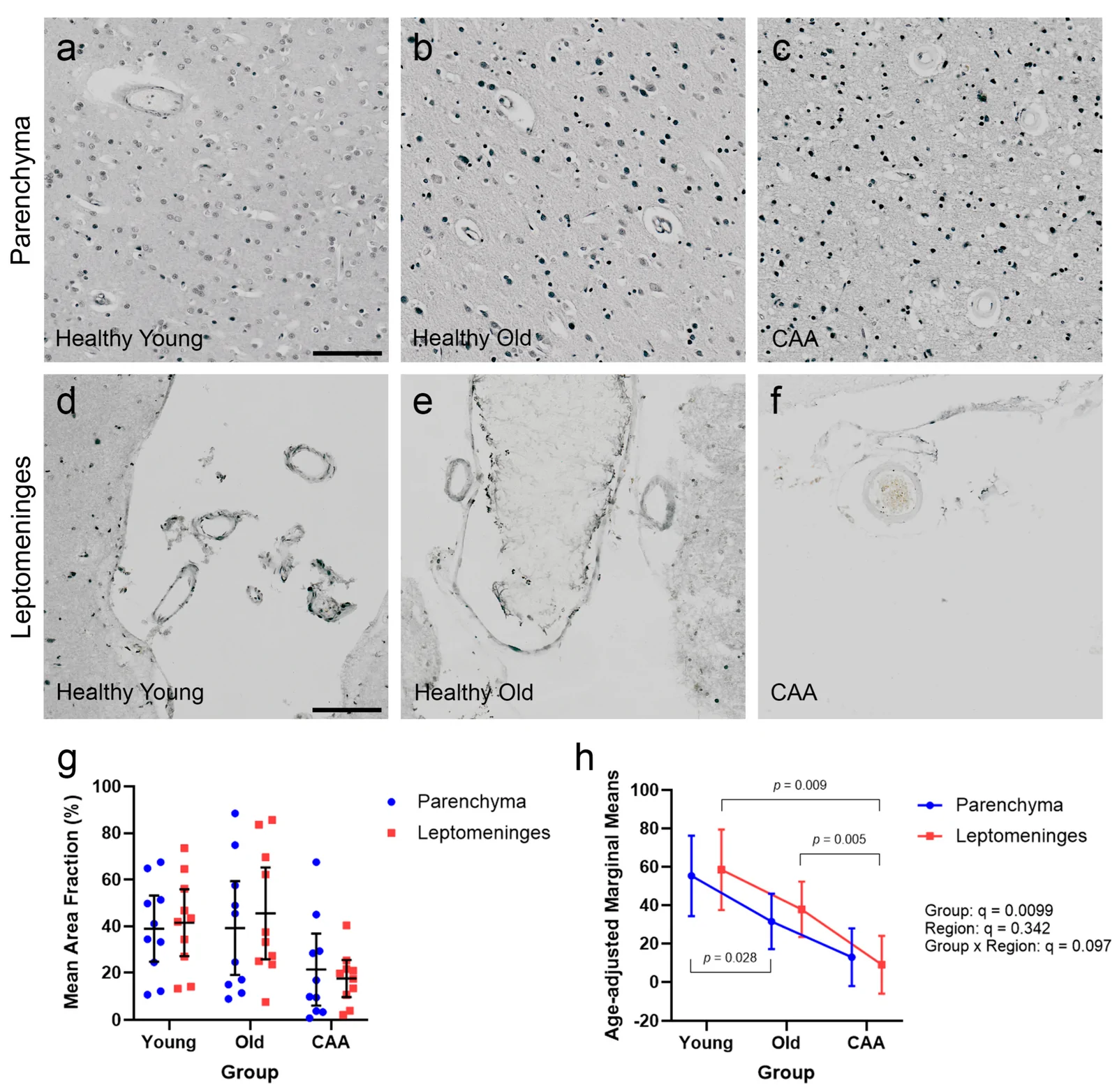
ADRA2B receptor immunoreactivity differs between diagnostic groups. Representative micrographs demonstrating immunoreactivity of ADRA2B in the walls of blood vessels in the brain parenchyma of healthy young (**a**), healthy old (**b**), and CAA patients (**c**), and leptomeningeal vessels of healthy young (**d**), healthy old (**e**), and CAA patients (**f**). Scale bars = 100 µm. (**g**) Patient-level area fraction values. Each point represents the mean area fraction across five vessels selected from the indicated anatomical region of one donor; *n* = 10 donors per diagnostic group. (**h**) Interaction plot showing age-adjusted estimated marginal means and 95% confidence intervals from a linear mixed-effects model containing diagnostic group, anatomical region, age, and the group × anatomical region interaction as fixed effects, with donor identifier as a random intercept. The overall group effect was significant (q = 0.0099), whereas the group × anatomical region interaction was not (q = 0.097). Bonferroni-adjusted within-region comparisons identified differences between healthy young and CAA donors in parenchymal (*p* = 0.028) and leptomeningeal vessels (*p* = 0.009), and between healthy older and CAA donors in leptomeningeal vessels (*p* = 0.005). These contrasts should not be interpreted as evidence that diagnostic-group effects differed significantly between anatomical regions.

**Figure 4:**
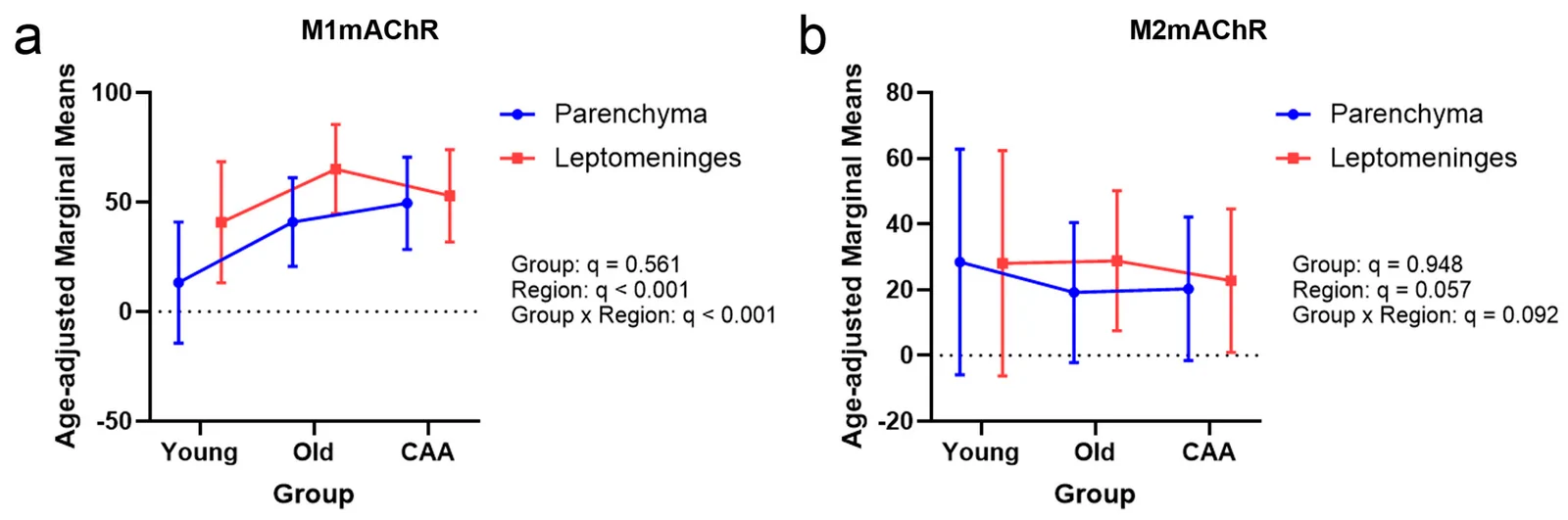
M1mAChR and M2mAChR receptor immunoreactivity in leptomeningeal and parenchymal vessels. Interaction plots showing (**a**) M1mAChR and (**b**) M2mAChR age-adjusted estimated marginal means and 95% confidence intervals from a linear mixed-effects model containing diagnostic group, anatomical region, age, and the group × anatomical region interaction as fixed effects, with donor identifier as a random intercept. The BH-FDR-adjusted main effect of anatomical region was significant for M1mAChR (*p* < 0.001) immunoreactivity. Significant group × anatomical region interactions were also observed for M1mAChR (*p* < 0.001), indicating that regional differences varied according to diagnostic group. No significant main effect of diagnostic group was detected for either receptor. Pairwise Bonferroni-adjusted comparisons within group × anatomical region combinations were not statistically significant.

**Figure 5:**
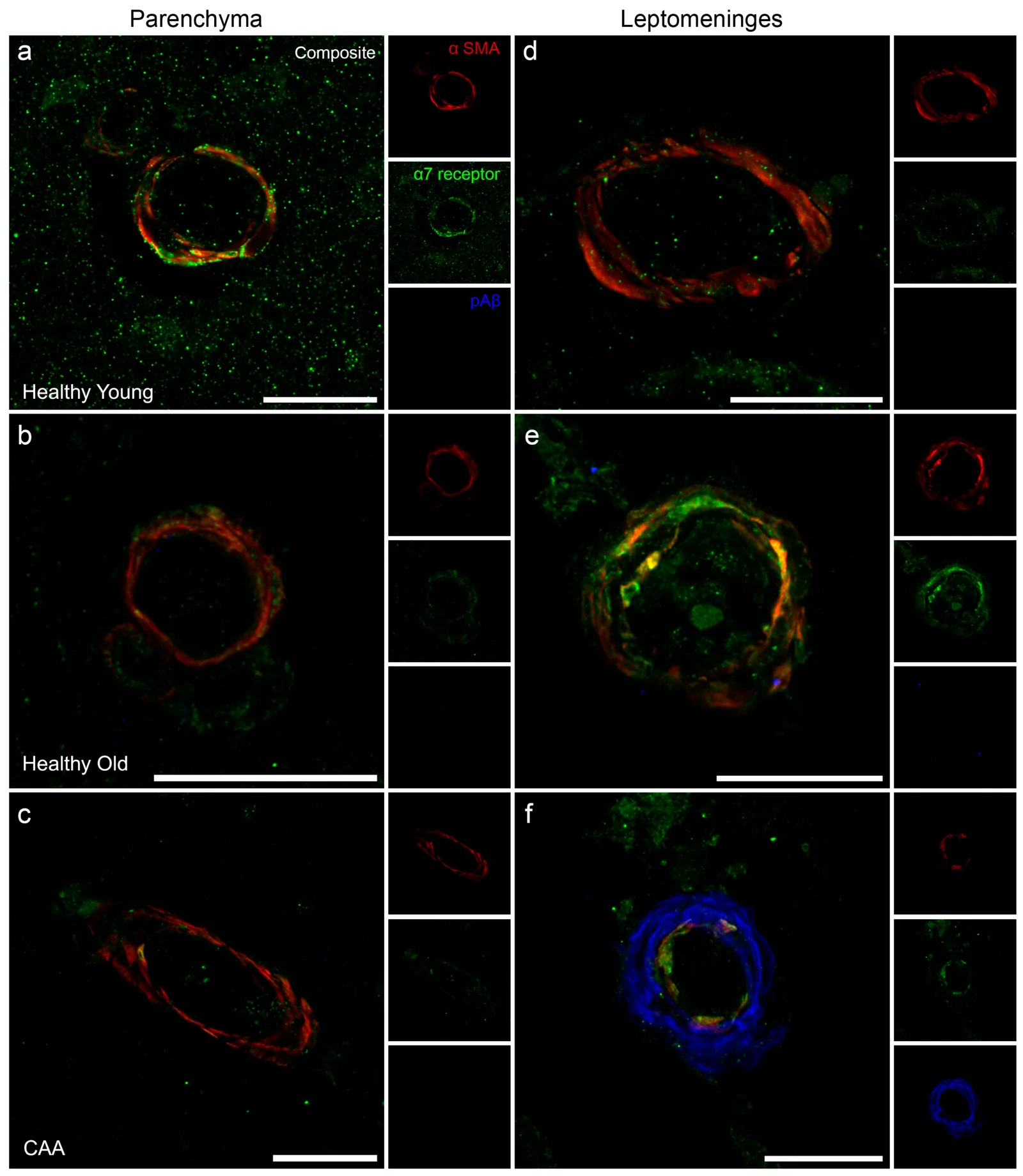
pAβ appeared to be qualitatively associated with α7nAChR-positive arterioles in CAA patients. Confocal micrographs showing αSMA (red), α7nAChR (green), and pAβ (blue) immunofluorescence in parenchymal (panels (**a**–**c**)) and leptomeningeal vessels (panels (**d**–**f**)) of healthy young, healthy old, and CAA patients. α7nAChR punctate staining shows qualitative association with smooth muscle cells and appears comparatively lower in healthy old and CAA patients. pAβ is largely absent in non-CAA patients, while being qualitatively associated with leptomeningeal vessels of CAA patient samples. Scale bars = 25 µm.

**Figure 6:**
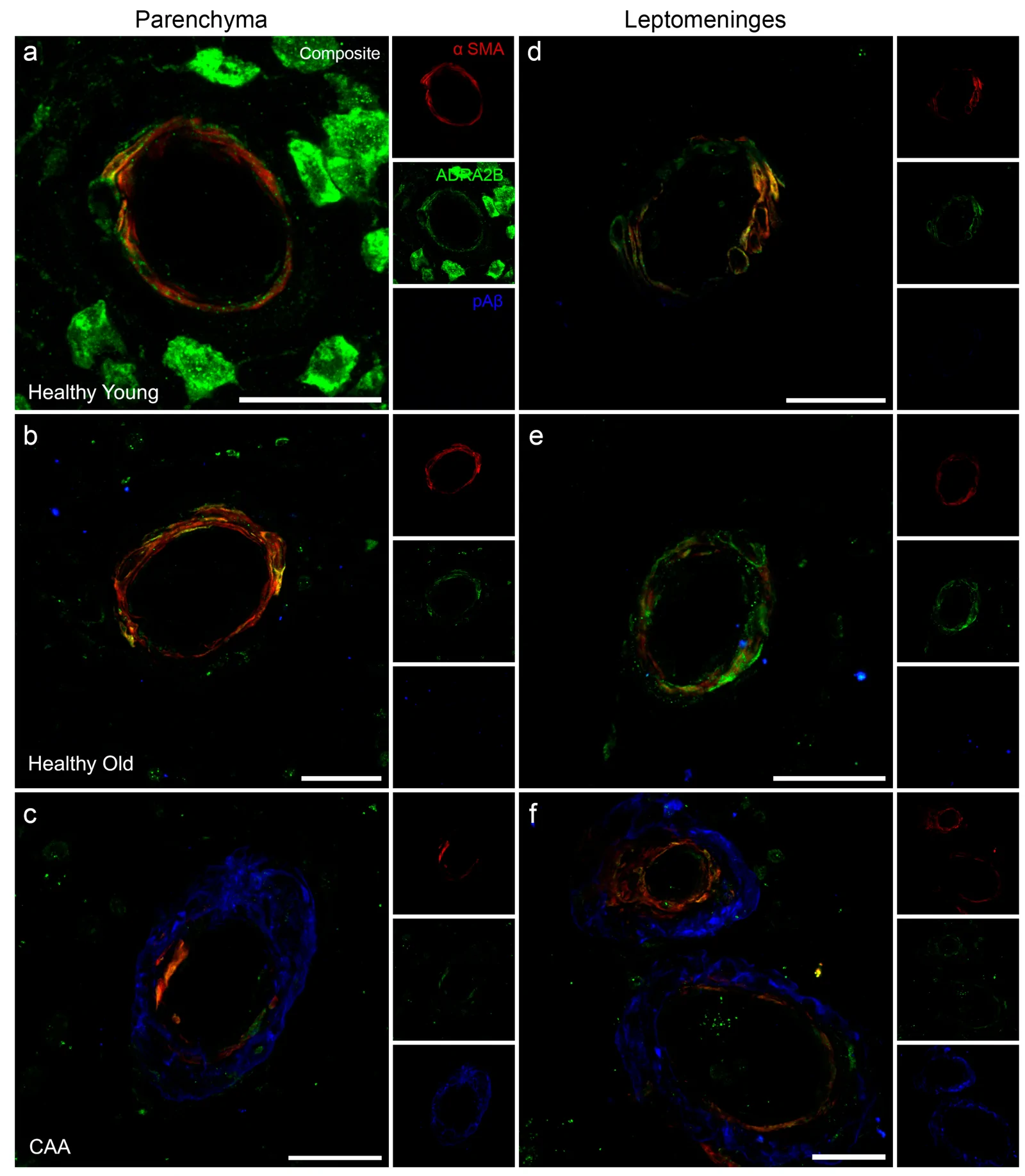
pAβ was spatially observed in ADRA2B-positive arterioles in CAA patients. Confocal micrographs showing αSMA (red), ADRA2B (green), and pAβ (blue) immunofluorescence in parenchymal (panels (**a**–**c**)) and leptomeningeal vessels (panels (**d**–**f**)) of healthy young, healthy old, and CAA patients. ADRA2B staining associated with smooth muscle cells is less pronounced than the ADRA2B-positive cells in the brain parenchyma (**a**). pAβ immunoreactivity is extensive in CAA patient groups in both parenchymal and leptomeningeal vessels. Scale bars = 25 µm.

**Table 1:** Raw and Benjamini–Hochberg FDR-adjusted omnibus probabilities for all five receptor models.

Receptor	Group		Anatomical Region		Group × Region	
	*p*-Value	BH-FDR-Adjusted q-Value	*p*-Value	BH-FDR-Adjusted q-Value	*p*-Value	BH-FDR-Adjusted q-Value
α7nAChR	0.003605	0.009862834	1.94 × 10^−7^	4.84075 × 10^−7^	1.32 × 10^−5^	2.20203 × 10^−5^
ADRA2B	0.003945	0.009862834	0.341964	0.341964029	0.097243	0.097243315
ADRA1B	0.117571	0.195952082	0.171794	0.214742611	6.1 × 10^−6^	1.52497 × 10^−5^
M1mAChR	0.448826	0.561032697	4.53 × 10^−17^	2.2634 × 10^−16^	4.73 × 10^−6^	1.52497 × 10^−5^
M2mAChR	0.947777	0.947776634	0.033969	0.056614993	0.073616	0.092019732

**Table 2:** Primary and secondary antibodies used for DAB staining. HIER = Heat-induced epitope retrieval.

Primary Antibody	Supplier	Dilution	Secondary Antibody	Supplier	Dilution	Antigen Retrieval
α7 nicotinic acetylcholine	Biotechne rabbit NBP1-79948	1/400	biotinylated goat anti-rabbit	Vector Laboratories BA-1000	1/400	HIER
Muscarinic M1	Alomone rabbit AMR 001	1/500	biotinylated goat anti-rabbit	Vector Laboratories BA-1000	1/400	HIER
Muscarinic M2	Alomone rabbit AMR 002	1/200	biotinylated goat anti-rabbit	Vector Laboratories BA-1000	1/400	HIER, pepsin
Muscarinic M3	Alomone rabbit AMR 003	1/400	biotinylated goat anti-rabbit	Vector Laboratories BA-1000	1/400	HIER, pepsin
Adrenergic α1b	Proteintech rabbit 19776-1-AP	1/500	biotinylated goat anti-rabbit	Vector Laboratories BA-1000	1/400	HIER, pepsin
Adrenergic α2b	Proteintech rabbit 19778-AP	1/300	biotinylated goat anti-rabbit	Vector Laboratories BA-1000	1/400	HIER

**Table 3:** Primary and secondary antibodies used for immunofluorescence. HIER = Heat-induced epitope retrieval.

Primary Antibody	Supplier	Dilution	Secondary Antibody	Supplier	Dilution	Antigen Retrieval
Smooth muscle actin	Sigma mouse 3777 FITC	1/200	None (FITC conjugated primary)	-	-	HIER
Adrenergic/cholinergic receptors	See Table 1	See Table 1	Alexa Fluor 555^TM^	Thermo Fisher A-21428	1/200	HIER, pepsin
Abeta-pE3	Synaptic Systems mouse 218011	1/400	Alexa Fluor 647^TM^	Thermo Fisher, A-21240	1/200	HIER, pepsin

## Data Availability

The data that support these findings are openly available on Figshare at https://figshare.com/; under the reference number https://doi.org/10.6084/m9.figshare.32658156. Further inquiries can be directed to the corresponding authors.

## Supplementary Materials

The following supporting information can be downloaded at: https://www.mdpi.com/article/10.3390/ph19101518/s1, Figure S1: Human patient age distributions; Figure S2: Antibody validation; Figure S3: Image acquisition; Figure S4: ImageJ macro for analysis of receptor immunoreactivity in cerebral blood vessels; Figure S5: Image processing; Table S1: Human Tissue Details; Table S2: Thresholds for image analysis.

## Abbreviations

The following abbreviations are used in this manuscript:

CAA	Cerebral Amyloid Angiopathy
AD	Alzheimer’s disease
Aβ	Amyloid-beta
IPAD	Intramural Periarterial Drainage
CSF	Cerebrospinal Fluid
LC	Locus Coeruleus
NBM	Nucleus Basalis of Meynert
pAβ	Pyroglutamylated amyloid-beta
α7nAChR	Alpha-7 Nicotinic Acetylcholine Receptor
M1mAChR	M1 Muscarinic Acetylcholine Receptor
M2mAChR	M2Muscarinic Acetylcholine Receptor
M3mAChR	M3Muscarinic Acetylcholine Receptor
ADRA1B	Alpha-1BAdrenergic Receptor
ADRA2A	Alpha-2A Adrenergic Receptor
ADRA2B	Alpha-2BAdrenergic Receptor
