# Supplementary material for: Changes in Adrenergic and Cholinergic Receptors on Human Cerebral Vessels
Source: Pharmaceuticals (Basel). 2026 Sep 25;19(10):1518. doi: 10.3390/ph19101518 (PMC13708240; doi:10.3390/ph19101518)
Supplement: Supplementary file 1 [file pharmaceuticals-19-01518-s001.zip › pharmaceuticals-4431041-supplementary.pdf]

Supplementary Material

A

	Young	Old	CAA
**Mean age (years)**	37.86	81.71	80.38
**SD**	13.5	10.44	6.82
**n**	28	24	26

B

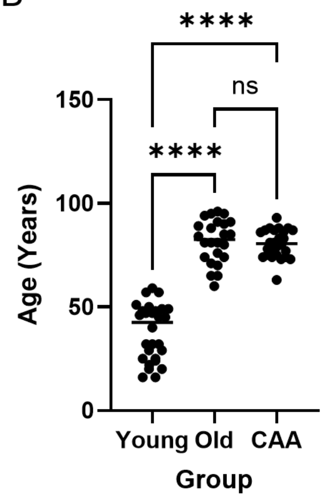

Figure S1: Human patient age distributions. (A) Mean age, standard deviation (SD), and total group size (n) for each diagnostic group. (**B**) Scatter plot of ages for each patient in healthy young, healthy old, and CAA groups. One-way ANOVA analysis with pairwise comparisons showed that the mean age of the healthy young group is significantly different to the mean ages of the healthy old and CAA groups. The mean ages of the healthy old and CAA groups were not significantly different.

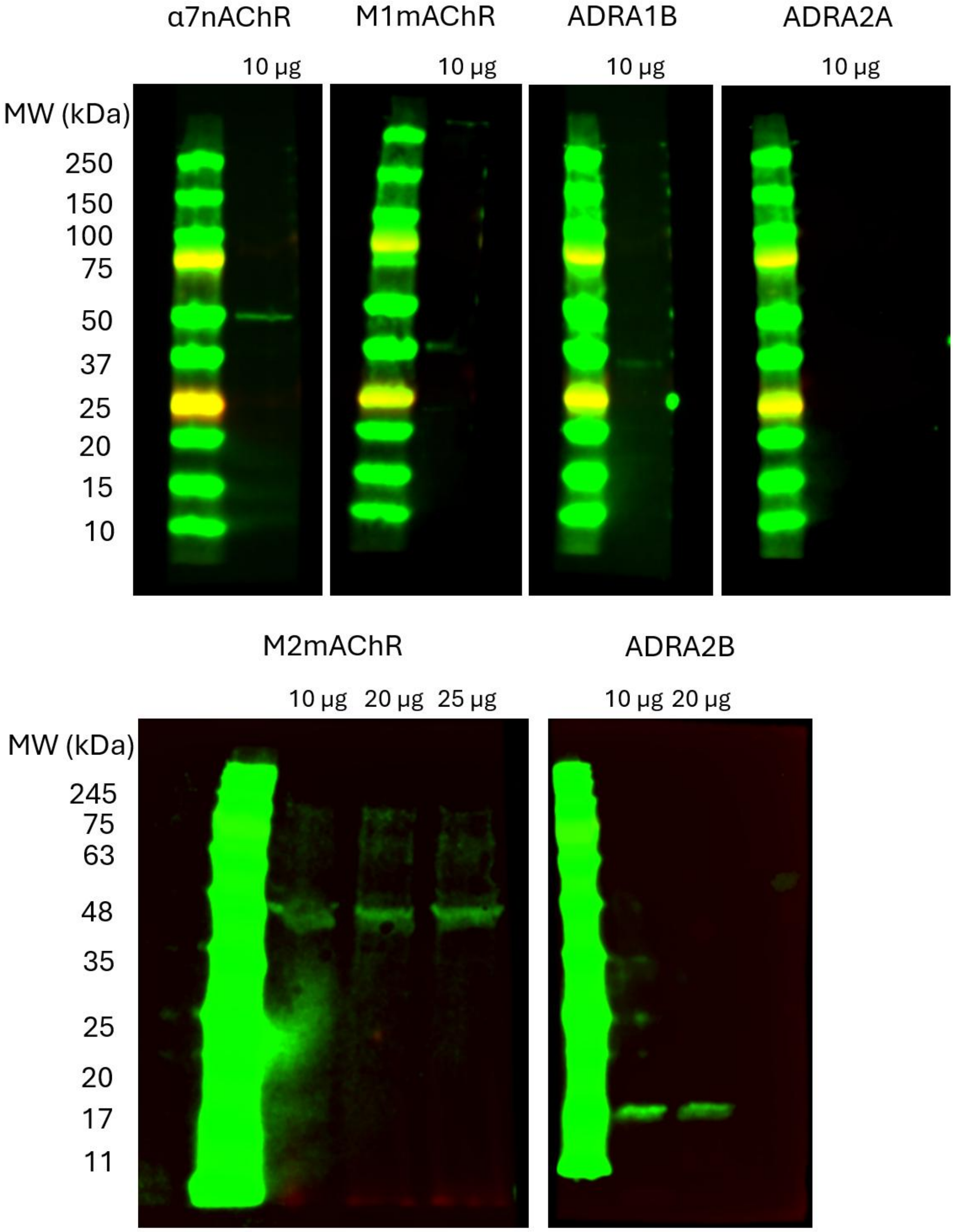

Figure S2: Antibody validation. Western blot images of primary human smooth muscle cell lysates separated on 12% TGX gels and probed with primary antibodies against six different receptors at 1:200 dilution. All antibodies except ADRA2A produced bands.

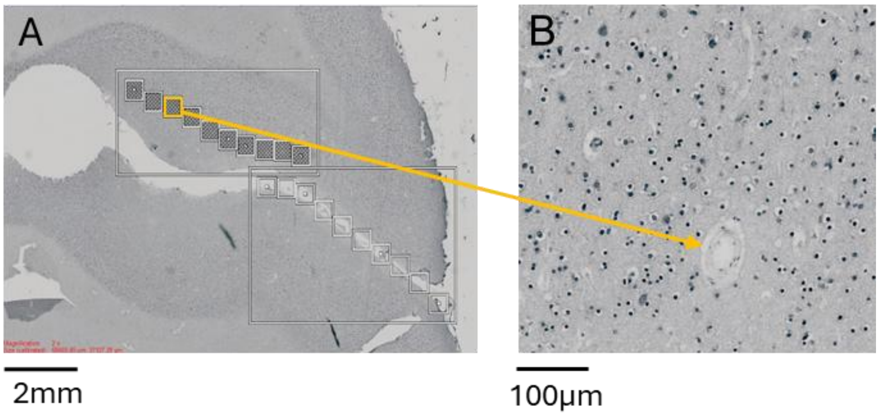

Figure S3: Image acquisition. (**A**) Tissue microarray core placement in grey matter and sulcus. (**B**) Representative x20 scan of the cores.

Figure S4. ImageJ macro for analysis of receptor immunoreactivity in cerebral blood vessels.

```
title=getTitle();
run("Set Scale...", "distance=3.1 known=1 unit=Âµm");
run("8-bit");
run("Grays");
run("Invert");
run("Enhance Contrast", "saturated=0.35");
title1 = "draw polygon around arteriole, try to avoid surrounding tissue, press ok";
waitForUser(title1);
run("Clear Outside");
title2 = "draw polygon around arteriole lumen, press ok";
waitForUser(title2);
run("Clear", "slice");
title3 = "click outside the polygon so that it disappears, press ok";
waitForUser(title3);
setThreshold(1, 255, "raw");
run("Analyze Particles...", "size=0-Infinity pixel display clear summarize add");
setThreshold(118, 255, "raw");
run("Analyze Particles...", "size=0-Infinity pixel display clear summarize add");
selectWindow("Summary");
```

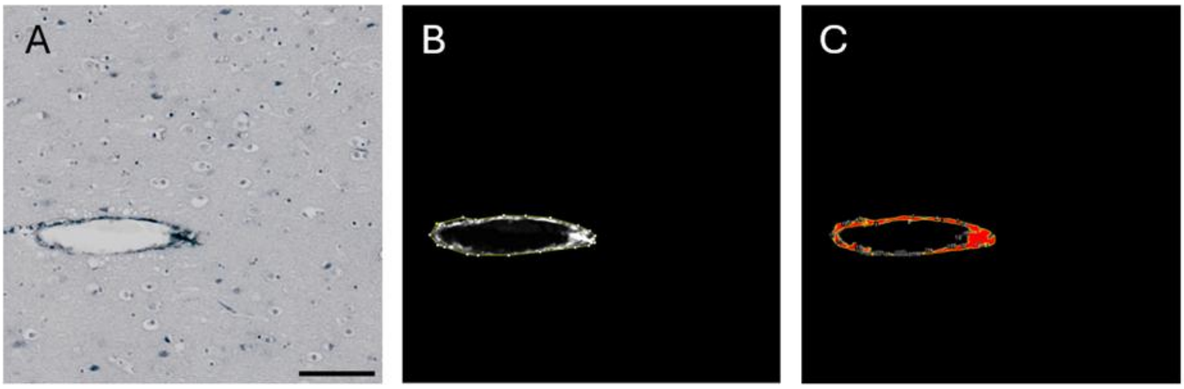

Figure S5. Image processing. Micrographs were processed using an ImageJ macro (**A**). Blood vessels were manually outlined using the polygon tool and the background and lumen were removed to create a mask of the vessel (**B**). The total area of the vessel mask and pixels above the threshold for positive immunostaining were measured using the analyse particles tool (**C**). The ratio of these two values was used to calculate the % area of the vessel that contained immunoreactivity for each receptor. Scale bar = 100 µm

Table S1. Human Tissue Details

Sample ID	Sex	Age	Post-mortem delay (hrs)	Diagnosis	Group	Source
SD001-06	M	16	47	None	Healthy Young	Edinburgh Sudden Death Tissue Brain Bank
SD005-15	F	46	76	None	Healthy Young	Edinburgh Sudden Death Tissue Brain Bank
SD006-10	F	32	63	None	Healthy Young	Edinburgh Sudden Death Tissue Brain Bank
SD008-12	M	25	25	None	Healthy Young	Edinburgh Sudden Death Tissue Brain Bank
SD002-17	F	49	94	None	Healthy Young	Edinburgh Sudden Death Tissue Brain Bank
SD022-17	M	48	58	None	Healthy Young	Edinburgh Sudden Death Tissue Brain Bank
SD031-15	F	40	89	None	Healthy Young	Edinburgh Sudden Death Tissue Brain Bank
SD004-15	M	50	41	None	Healthy Young	Edinburgh Sudden Death Tissue Brain Bank
SD042-12	M	29	44	Small Vessel Disease	Healthy Young	Edinburgh Sudden Death Tissue Brain Bank
SD006-10	F	32	63	None	Healthy Young	Edinburgh Sudden Death Tissue Brain Bank
SD008-12	M	25	53	None	Healthy Young	Edinburgh Sudden Death Tissue Brain Bank
SD010-15	F	47	113	None	Healthy Young	Edinburgh Sudden Death Tissue Brain Bank
SD012-15	M	45	51	None	Healthy Young	Edinburgh Sudden Death Tissue Brain Bank
SD015-16	F	49	54	None	Healthy Young	Edinburgh Sudden Death Tissue Brain Bank
SD019-06	M	20	36	None	Healthy Young	Edinburgh Sudden Death Tissue Brain Bank
SD020-07	F	22	44	None	Healthy Young	Edinburgh Sudden Death Tissue Brain Bank
SD022-13	M	45	74	None	Healthy Young	Edinburgh Sudden Death Tissue Brain Bank
SD022-17	M	48	58	None	Healthy Young	Edinburgh Sudden Death Tissue Brain Bank

SD023-08	F	24	47	None	Healthy Young	Edinburgh Sudden Death Tissue Brain Bank
SD033-16	M	16	110	None	Healthy Young	Edinburgh Sudden Death Tissue Brain Bank
SD035-09	M	32	99	None	Healthy Young	Edinburgh Sudden Death Tissue Brain Bank
SD035-14	F	59	53	None	Healthy Young	Edinburgh Sudden Death Tissue Brain Bank
SD036-08	F	20	40	None	Healthy Young	Edinburgh Sudden Death Tissue Brain Bank
SD036-14	M	51	71	None	Healthy Young	Edinburgh Sudden Death Tissue Brain Bank
SD042-12	M	29	44	None	Healthy Young	Edinburgh Sudden Death Tissue Brain Bank
SD043-16	M	47	67	None	Healthy Young	Edinburgh Sudden Death Tissue Brain Bank
SD045-17	F	57	73	None	Healthy Young	Edinburgh Sudden Death Tissue Brain Bank
SD051-16	M	57	110	None	Healthy Young	Edinburgh Sudden Death Tissue Brain Bank
2008_109	M	81	52	DP	Healthy Old	Newcastle Brain Tissue Resource
2009_43	F	96	114	D-	Healthy Old	Newcastle Brain Tissue Resource
2010_685	F	88	22	CA	Healthy Old	Newcastle Brain Tissue Resource
2010_729	M	70	72	CA	Healthy Old	Newcastle Brain Tissue Resource
2011_1135	F	76	41		Healthy Old	Newcastle Brain Tissue Resource
2011_736	F	81	31		Healthy Old	Newcastle Brain Tissue Resource
2012_322	M	90	18		Healthy Old	Newcastle Brain Tissue Resource
2012_324	M	85	50		Healthy Old	Newcastle Brain Tissue Resource
2012_340	M	95	21	DS	Healthy Old	Newcastle Brain Tissue Resource
2012_531	M	74	70	PSP	Healthy Old	Newcastle Brain Tissue Resource
2013_400	F	65	47		Healthy Old	Newcastle Brain Tissue Resource
2014_496	F	80	31		Healthy Old	Newcastle Brain Tissue Resource
2015_2907	M	94	25		Healthy Old	Newcastle Brain Tissue Resource
2015_2910	M	91	24		Healthy Old	Newcastle Brain Tissue Resource
2015_2922	F	84	72		Healthy Old	Newcastle Brain Tissue Resource
2015_365	M	91	72		Healthy Old	Newcastle Brain Tissue Resource
2015_535	M	71	25		Healthy Old	Newcastle Brain Tissue Resource
2016_315	F	65	16		Healthy Old	Newcastle Brain Tissue Resource
2016_522	M	60	60		Healthy Old	Newcastle Brain Tissue Resource
324_12	M	85	50		Healthy Old	Newcastle Brain Tissue Resource
477_11	F	95	66		Healthy Old	Newcastle Brain Tissue Resource
531_12	M	74	70		Healthy Old	Newcastle Brain Tissue Resource
648_11	F	89	98		Healthy Old	Newcastle Brain Tissue Resource
736_11	F	81	31		Healthy Old	Newcastle Brain Tissue Resource
1029_12				Severe CAA	CAA	Newcastle Brain Tissue Resource
2009_02	F	77	63	Severe CAA	CAA	Newcastle Brain Tissue Resource
2009_04	M	79	13	Severe CAA	CAA	Newcastle Brain Tissue Resource

2009_51	F	73	47	Severe CAA	CAA	Newcastle Brain Tissue Resource
2009_65	M	75	82	Severe CAA	CAA	Newcastle Brain Tissue Resource
2010_1115	M	77	78	Severe CAA	CAA	Newcastle Brain Tissue Resource
2010_320	M	88	84	Severe CAA	CAA	Newcastle Brain Tissue Resource
2010_725	F	87	54	Severe CAA	CAA	Newcastle Brain Tissue Resource
2011_0715	M	83	71	Severe CAA	CAA	Newcastle Brain Tissue Resource
2011_088	F	88	84	Severe CAA	CAA	Newcastle Brain Tissue Resource
2011_1016	F	74	49	Severe CAA	CAA	Newcastle Brain Tissue Resource
2011_715	M	83	71	Severe CAA	CAA	Newcastle Brain Tissue Resource
2012_151	F	86	51	Severe CAA	CAA	Newcastle Brain Tissue Resource
2012_580	M	73	7	Severe CAA	CAA	Newcastle Brain Tissue Resource
2013_045	M	81	41	Severe CAA	CAA	Newcastle Brain Tissue Resource
2013_157	M	80	39	Severe CAA	CAA	Newcastle Brain Tissue Resource
2013_1902	F	81	83	Severe CAA	CAA	Newcastle Brain Tissue Resource
2014_388	M	87	18	Severe CAA	CAA	Newcastle Brain Tissue Resource
2015_2901	F	74	99	Severe CAA	CAA	Newcastle Brain Tissue Resource
2015_2903	F	93	12	Severe CAA	CAA	Newcastle Brain Tissue Resource
2017_4929	M	63	59	Severe CAA	CAA	Newcastle Brain Tissue Resource
320_10	M	88	84	Severe CAA	CAA	Newcastle Brain Tissue Resource
505_10	F	86	47	Severe CAA	CAA	Newcastle Brain Tissue Resource
530_12				Severe CAA	CAA	Newcastle Brain Tissue Resource
65_09	M	75	82	Severe CAA	CAA	Newcastle Brain Tissue Resource
67_09	F	74	67	Severe CAA	CAA	Newcastle Brain Tissue Resource

Table S2. Thresholds for image analysis.

Receptor	Parenchyma	Leptomeninges
α7 nicotinic receptor	107	102
M1 Muscarinic receptor	105	106
M2 Muscarinic receptor	105	102
ADRA1b	129	122
ADRA2a	116	105
ADRA2b	109	104
